# Childhood injuries and food stamp benefits: an examination of administrative data in one US state

**DOI:** 10.1186/s12887-020-02084-y

**Published:** 2020-06-17

**Authors:** Colleen M. Heflin, Irma Arteaga, Jean Felix Ndashimye, Matthew P. Rabbitt

**Affiliations:** 1 Public Administration and International Affairs, Syracuse University, 426 Eggers Hall, Syracuse, NY 13244-1020 USA; 2 University of Missouri, Columbia, USA; 3 Vanderbilt University, Nashville, USA; 4 USDA, Economic Research Service, Kansas City, MO, USA

**Keywords:** Childhood injury, Food stamps, Food insecurity

## Abstract

**Background:**

Currently in the United States, childhood injuries are the leading cause of mortality and morbidity, resulting in an estimated 9.2 million emergency department visits and $17 billion annually in medical costs. For preschoolers, it is also the leading cause of disability.

**Methods:**

We use linked administrative data for SNAP and Medicaid in Missouri from January 2010 to December 2013 to explore monthly patterns in the association between SNAP receipt and ER claims due to childhood injury for children age 0–5 and to examine if these patterns are sensitive to the timing of SNAP benefits. We chose the state of Missouri because unlike most states that disburse SNAP benefits within the first 10 days of the calendar month, Missouri pays SNAP benefits between the first twenty-two days of the month, based on the recipient’s birthdate and last name.

**Results:**

SNAP benefits received later in the calendar month are associated with reductions in ER claims for childhood injuries. Furthermore, the final week in the SNAP benefit month is associated with an increase in ER claims for childhood injuries.

**Conclusion:**

In terms of public policy, our results suggest that having SNAP disbursement later in the month may have benefits for households.

## Background

Currently in the United States, childhood injuries are the leading cause of mortality and morbidity, resulting in an estimated 9.2 million emergency department visits and $17 billion annually in medical costs [2]. For preschoolers, it is also the leading cause of disability [10]. Poverty is a risk factor for experiencing childhood injuries and injuries are more prevalent among low-income families [1, 27, 4]. Besides demographic risk factors, other known correlates of childhood injury tend to focus on diminished parenting practices [10, 19, 23–25, 32] and increased child behavior problems [8], which are also correlated with maternal stress and income. For children under 15, the unintentional injury mortality rate was the highest among those less than 1 year of age, followed by children 1 to 4 years of age [2]. While the overall unintentional mortality injury rate for the US is 15.0 per 100,000, for the state of Missouri, this rate is 21.2 per 100,000. Previous research suggests that food insecurity is likely to be a catalyst for many of the specific mechanisms that increase the likelihood of childhood injury [3, 33]. This study explores the extent to which food insecurity, or the inconsistent access to food through socially acceptable ways, is associated with temporal patterns in ER visits for childhood injuries among children less than 5 years of age in the state of Missouri.

Our study population is comprised of children age 0–5 who participated in both Medicaid and the Supplemental Nutrition Assistance Program (SNAP), a federal nutritional program that provides food assistance in the form of vouchers with an average household value of $255 to purchase food products, seeds, and plants that produce food for consumption to low-income individuals. SNAP is the largest food assistance program administered by the US Department of Agriculture, serving 18 million children (one in four children) in 2016 [7]. Sixty-nine percent of children in households receiving SNAP are school aged (age 5 to 17) and 55% of all households with children earned any income [30]. According to one estimate, over the entire childhood period, nearly half of all children will reside in a household that receives food stamps at some point [3, 26].

Households are income eligible for SNAP if they have a gross monthly income below 130% of the federal poverty line or, by state option, through participation in another low-income assistance program. Furthermore, children in Missouri are eligible for public health insurance if they reside in households with household incomes below 148% of the federal poverty line [21], meaning that all children in households who are income-eligible for SNAP benefits are also income-eligible for Medicaid.

**Figure 1:**
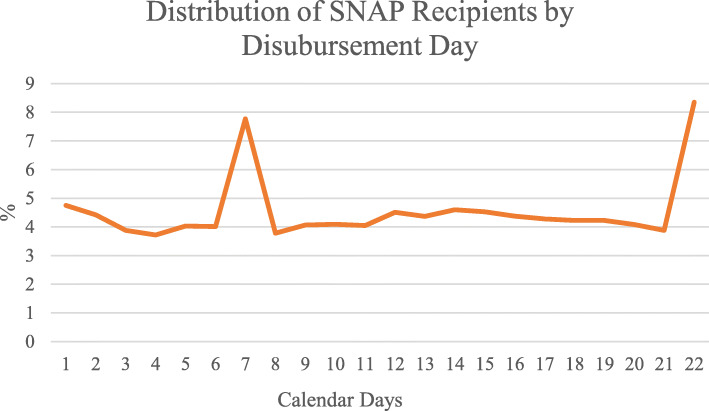
Fig. 1 Percentage of SNAP Benefits Disbursed by Calendar Day 1-22

**Figure 2:**
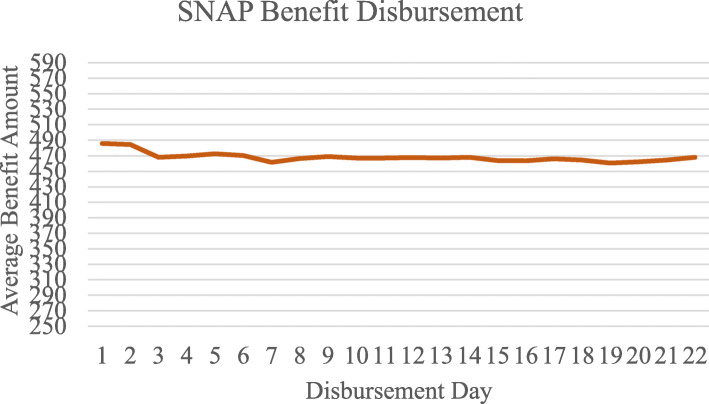
Fig. 2  Average SNAP Benefit Amount Disbursed by Calendar Day 1-22

The federal government provides states with flexibility in deciding how they disburse benefits. While many states choose to disburse SNAP benefits within the first 9 days of the calendar month, at the time of this study Missouri was the only state in the country to issue SNAP benefits between the 1st and 22nd day of the month, with the specific calendar day assignment based on a combination of the case head’s first letter of the last name and their birth month. For example, on the 1st everyone born in January with last name A-K receives benefits, on the 2nd everyone born in January with last name L-Z receive benefits, and on the 3rd everyone born in February with last name A–K receives benefits [31]. Since the date of SNAP benefit payment varies across households according to rules that are outside the control of individual decisions or outcomes, these data are ideal for examining the causal impact of benefit payment timing. As we observe in Figures 1 and 2 in Appendix, the percentage of SNAP recipients receiving SNAP disbursement, as well as the average amount of benefits receiving is very similar through the calendar month (from day 1 through 22). This demonstrates the exogenous variation in the SNAP disbursement data used to identify our models.

The introduction of SNAP is associated with positive improvements in child outcomes [18]. However, there is also evidence that because SNAP benefits are often exhausted well before the next issuance date, household food consumption may be inconsistent as households deplete their resources ( [5, 6, 14, 29]. National estimates suggest that 60% of SNAP households use all of their benefits in the week following benefit disbursement and 91% of households use all of their benefits in the first 3 weeks following benefit disbursement.

In this paper, we examine how SNAP participation affects patterns in ER visits for childhood injuries. We hypothesize that among households that receive SNAP, depletion of benefits over the month might increase childhood injuries near the end of the month as both child and parental behaviors deteriorate under the strain of food insecurity. Several theoretical arguments support this hypothesis. First, prior research has documented that there are monthly patterns to the SNAP benefit exhaustion [6, 15], and that households reduce food intake at the end of the benefit month because of financial hardship [34]. Further research finds that food spending decreases toward the end of the calendar month among SNAP households that receive benefits early in the calendar month but not among SNAP households that receive benefits later in the calendar month [9]. Thus, previous research has linked the distribution of SNAP benefits to within month cycles in food insecurity.

Secondly, previous research has linked the timing of household SNAP benefit receipt to children’s test scores and negative behaviors [11–13]. Given the focus in the childhood injury literature on diminished parenting such as not coping with parenthood well, low rule enforcement, lack of supervision, low levels of every day routines, and maternal fatigue [10, 19, 23–25, 32] as well as increased child behavior problems [8], we examine the relationship between timing of SNAP benefits and Medicaid claims for ER visits for childhood injuries using data from Missouri. By combining state administrative data from two programs, this study makes an important contribution to our understanding of the social and environmental predictors of flare-ups of childhood injuries while also exploring the consequences of specific implementation choices related to food and nutrition policies, such as the date of SNAP benefit disbursement.

## Methods

We use state administrative data from the Missouri Department of Social Services for SNAP program services from January 2010 to December 2013 linked to Medicaid claims data for children age 0 to 5 for the same time period. A total of 1,288,552 Medicaid claims were submitted for emergency care in Missouri for children age 0 to 5 living in a household that received SNAP. Our analysis, thus, is limited in that we can only identify injuries among the sample of children who submit ER claims to Medicaid.

In order to identify ER care due to an injury, we used International Classification of Disease, Ninth Revisions (ICD-9) diagnosis codes 800–999 as well as those for Abuse Head Trauma injuries (781.0–781.4, 781.8) [20].^1^ We coded cases as injuries if the ICD-9 code indicated that an injury was present among the first five diagnoses for a patient on a given day. In total, we were able to identify 260,907 ER claims as being injury-related condition; out of every 100,000 childhood ER claims submitted to Medicaid 20,250 involved an injury-related condition.

As described elsewhere [17, 28], we analyze the relationship between SNAP issuance and Medicaid ER claims for chilhood injuries by creating: 1) a standardized 28 day calendar month and 2) a standardized 28 day SNAP benefit month, where SNAP benefit month begins on the first day of SNAP receipt. We also include the set of demographic characteristics contained in SNAP administrative records-- sex, age, race, household size, and Hispanic ethnicity. In addition, we also include dummy variables indicating the calendar year to control for policy and economic changes over the time period. See Table 4 in Appendix for descriptive statistics on our study population.

Our research hypothesis is that children in households receiving SNAP benefits may (following exhausting of SNAP benefits) experience periods of extreme stress related to their inability to meet basic needs in which both child and parenting behavior deteriorates and make child injuries more likely. We might see evidence of the SNAP benefit pay cycle as an important determinant of injury-related ER claims if SNAP supports food consumption mainly. Alternatively, we might observe evidence that SNAP helps free up resources for other expenditures if SNAP benefits received later in the calendar month are more helpful at smoothing family functioning than SNAP benefits received earlier in the calendar month. We examine both of these possible hypotheses separately.^2^ We present average marginal effects from probit models for childhood injury claims in weeks 2, 3 and 4 (week 1 is omitted) for first the calendar month week and then the SNAP benefit month week with heteroscedastic robust standard errors which are clustered at the individual level to account for multiple observations per child.

We begin by estimating the probability of a childhood injury claim as a function of the calendar week for the full sample regardless of SNAP disbursement date. Then, we split the sample of ER claims by the week of SNAP benefit receipt. Results between SNAP receipt and injury-related ER claims in the set of states that disburse SNAP benefits in the first week of the month are proxied in our data by the set of households who receive their SNAP in the first calendar week. Whereas, our results in calendar weeks 2 and 3 provides an indication of how a staggered SNAP disbursement schedule may influence childhood injury-related healthcare utilization patterns.

We test the sensitivity of our main result to two different specifications first explored by Heflin et al. [16]. First, we split the sample of ER claims into those with and without any reported earnings to explore the possibility that households without earned income are more responsive to the issuance schedule of SNAP. Households without earnings likely have access to other means-tested social policy programs which disburse benefits at the beginning of the calendar month on a single date, such as TANF, the Special Supplemental Nutrition Program for Women, Infants, and Children (WIC), or Supplemental Security Income (SSI). As a consequence, SNAP benefits received in the last half of the month may have a larger affect in reducing childhood injuries by boosting food consumption at a point when other resources may be depleted and helping the family avoid stress that might trigger negative child or parenting behaviors associated with childhood injuries. In contrast, we expect that it is somewhat easier for households with multiple sources of income flowing into the household throughout the month to smooth their food consumption and provide a more supportive environment for children and parenting. According to national data from the US Department of Agriculture, just over half of SNAP households with children had earned income [30].

As a second sensitivity test, we split our sample by length of time of current SNAP spell of receipt (first or second observed month versus those who have been on SNAP for 3 months or longer). This analysis, as suggested by Heflin et al. [16] is a test of the hypothesis that food insecurity is associated with poor financial literacy skills and the inability budget resources over the month in order to smooth consumption and avoid periods of hardship. Accordingly, SNAP participants who are new to the program are most likely to be sensitive to the SNAP benefit month while those who have been on the program for several months might be better at smoothing consumption over the benefit month.

## Results

### Calendar month

We estimate the probability that a child age 0–5 in a SNAP household is observed submitting an injury-related ER claim to Medicaid based on the week of the calendar month, controlling for SNAP benefit amount and basic demographic characteristics in Table 1. In column 1, we present results for the pooled sample and observe that, contrary to our expectations, the probability for childhood injury claims is lowest in the last week of the calendar month.

**Table 1:** Average marginal effects of calendar week on probability of ER claims for childhood injuries overall and by week of SNAP benefit disbursement

Variable	Total Sample	Week of SNAP Benefit Disbursement		
		Week 1	Week 2	Week 3
Calendar Week 2	−0.0007	−0.0021	0.0022	−0.0022
	(0.0018)	(0.0030)	(0.0030)	(0.0030)
	[0.674]	[0.486]	[0.474]	[0.473]
Calendar Week 3	−0.0020	− 0.0002	− 0.0027	− 0.0029
	(0.0017)	(0.0030)	(0.0030)	(0.0030)
	[0.247\	[0.938]	[0.354]	[0.332]
Calendar Week 4	−0.0080***	− 0.0042	− 0.0037	−0.0161***
	(0.0018)	(0.0031)	(0.0031)	(0.0031)
	[0.000]	[0.185]	[0.224]	[0.000]
Observations	1,288,552	419,676	438,145	430,731

*** p < 0.01, ** p < 0.05, * p < 0.1

Source: Authors’ analysis of data from Missouri Department of Social Services. N=. 1,288,552

Notes: Results are from probit regression models controlling for SNAP benefit amount, race/ethnicity, sex, age, household size, and year of ER visit. Average marginal effects with standard errors in parentheses and p-values in brackets. Standard errors clustered at the individual level

In columns 2–4 of Table 1, we split the sample by the week of the calendar week of the SNAP disbursement. Since SNAP disbursement date assignment is close to random, we can infer that observed differences in the relationship between the calendar week of SNAP disbursement and the probability of childhood injury-related ER claims across columns 2–4 are a result of timing differences in receipt. While there is no association between the calendar week and childhood injury claims for those that receive their SNAP benefits in the first and second week, those who receive their benefits in week 3 have a reduction of childhood injuries claims in weeks 4.^3^

In Table 2, we split the sample by earned income status and examine the relationship between the timing of SNAP benefits and the calendar week of the ER claim to see if having access to other sources of income allows SNAP households to smooth their consumption more easily and avoid fluctuations in the risk of childhood injuries. Our results were similar to the ones found in Table 1. While, we observe that households with no earnings have a lower risk of submitting an ER claim in the last week of the calendar month in comparison to households with some earnings, the difference between these two effects was not statistically significant. Similarly, the risk of submitting an ER claim in other weeks of the calendar month was statistically similar for those with no earnings in comparison to those with some earnings.

**Table 2:** Average marginal effect of the probability of ER visit for childhood injury relative to calendar week 1 by earned income status

Variable	No Earned Income	Some Earned Income
Calendar Week 2	0.0005	−0.0025
	(0.0022)	(0.0028)
	[0.822]	[0.367]
Calendar Week 3	0.0008	−0.0061**
	(0.0022)	(0.0028)
	[0.730]	[0.028]
Calendar Week 4	−0.0082***	−0.0078***
	(0.0023)	(0.0029)
	[0.000]	[0.007]
Observations	767,252	521,300

*** p < 0.01, ** p < 0.05, * p < 0.1

Source: Authors’ analysis of data from Missouri Department of Social Services. N=. 1,288,552

Notes: Results are from probit regression models controlling for SNAP benefit amount, race/ethnicity, sex, age, household size, and year of ER visit. Average marginal effects with standard errors in parentheses and p-values in brackets. Standard errors clustered at the individual level. We ran a test to compare the statistical difference of the reported effects and compared column (1) with column (2). We did not find any statistical significance difference

### SNAP benefit month

In Table 3, we present analysis by the SNAP benefit month. Here, we are testing the hypothesis that households treat SNAP benefits differently from other financial resources and that their protective benefit can be observed over a month in which the first day of SNAP disbursement is considered to be day 1 of the SNAP benefit month, regardless of the calendar day in which it occurs. Results in Table 3 support the hypothesis that the last week of the SNAP benefit week is the week with the highest probability of submitting an ER claim for childhood injury (*p* = .046).

**Table 3:** Average marginal effects of SNAP benefit week on probability of ER claims for childhood injuries overall and by length of time on SNAP

Variable	Total Sample	Length on SNAP	
		New Users	Old Users
Benefit Week 2	−0.0022	−0.0052**	− 0.0008
	(0.0017)	(0.0026)	(0.0025)
	[0.207]	[0.042]	[0.763]
Benefit Week 3	0.0028	0.0006	0.0023
	(0.0018)	(0.0026)	(0.0026)
	[0.114]	[0.806]	[0.384]
Benefit Week 4	0.0036**	−0.0001	0.0051*
	(0.0018)	(0.0027)	(0.0026)
	[0.046]	[0.979]	[0.053]
Observations	1,288,552	596,364	580,066

*** p < 0.01, ** p < 0.05, * p < 0.1

Source: Authors’ analysis of data from Missouri Department of Social Services. N=. 1,288,552

Notes: Results are from probit regression models controlling for SNAP benefit amount, race/ethnicity, sex, age, household size, and year of ER visit. Average marginal effects with standard errors in parentheses and p-values in brackets. Standard errors clustered at the individual level. We ran a test to compare the statistical difference of the reported effects and compared column (1) with column (2). We did not find any statistical significance difference

In the right-hand side of Table 3, we present results with the sample split by those that are new to SNAP and may experience difficulty stretching their benefits over the entire month and those that have been on SNAP for 3 or more months and are hypothesized to be more likely to have adopted budgeting techniques to avoid disruptions in the monthly food supply. While it seems that new users to SNAP do not experience a higher probability of ER claims for childhood injuries at the end of the month, they do experience a decline in the 2nd week of the SNAP benefit week. However, when we compare our effects for new users to those with old users, the difference in the coefficients is not statistically significant. Similarly, old users of SNAP seem to have a higher probability of submitting an ER claim for childhood injuries in the last week of the SNAP benefit week (*p* = .053); however, when we compare this effect with the one found for new users, once again the difference is not statistically significant.

## Discussion

In a typical month, SNAP serves families with about 20 million low-income children. About a third of all children participating in SNAP are under the age of five [30]. Injury is the leading cause of mortality and morbidity for children in the US [1]. While the likelihood of an ER visit due to child injury is multidimensional, key gaps in the literature persist. In this study, we examine the relationship between SNAP participation and child injury related ER visits. We chose the state of Missouri because unlike most states that disburse SNAP benefits within the first 10 days of the calendar month, Missouri pays SNAP benefits over the first twenty-two days of the month, based on the recipient’s birthdate and last name. Consequently, time of SNAP payment is close to random.

Our results showed that SNAP benefits received later in the calendar month might be especially protective for childhood injuries in the final week of the month by reducing food insecurity and subsequently reduce household stress (see Table 1), although the size of this effect is substantively small. Our findings also support the hypothesis that the last week of the SNAP benefit month is the week with the highest probability of submitting a child-injury related ER claim (Table 3). Putting these findings together, our analysis suggests that those receiving SNAP payments later in the calendar month are less likely to go to the ER for child-injury related cases, and this is because of the nature of the recipient benefits cycle. Because other sources of income or welfare programs such as TANF and WIC are received on a single day at the beginning of the month, SNAP benefits received later in the month would boost food consumption at a point when other in-kind and financial resources have been exhausted, becoming more protective than if SNAP is received earlier in the month.

We also split our sample by earned income status, families with no earnings and families with earnings, and examined the relationship between timing of SNAP benefits and timing of ER claim but found no statistically significant differences between the estimates of both groups. While these results are not consistent with our general expectation, one reason might be that Missouri is a state that does not use a broad-based categorical eligibility for SNAP and, as such, households with some income have very low levels of income and are not substantially different from those with no income. This is contrast to other states like Nevada or Maryland where SNAP eligibility rules do not have asset tests and the gross income limit of TANF is 200% of FPL. These results suggest that the ER claims for childhood injuries for both groups are lower at the end of month than they are at the beginning.

Although this study does not specifically test the channels through which SNAP might affect child-injury related ER visits, due to data limitations, the literature suggests that food insecurity affects parental stress and practices, which in turn, is a predictor of child injuries [10, 22]. Further research should shed light on the exact mechanisms through which SNAP affects child-injury related ER visits.

While this study yielded significant results that are consistent with our initial expectations, at least four limitations should be noted. First, our analysis is constrained to those who received both SNAP and Medicaid and have a child-injury related ER claim. Thus, it is not possible to generalize the results to any child-injury related ER claim or any ER claims. Second, this analysis was conducted for the state of Missouri and we cannot generalize these results for other states that are not demographically similar. Third, we can only control for a small number of demographic characteristics and other confounders may well bias our results. Fourth, we rely on ICD-9 codes to measure childhood injuries but there likely is some misclassifications and reporting error present.

## Conclusion

In conclusion, states have discretion on the timing of SNAP disbursement, and, therefore, state benefit issuance schedules vary. Currently, only one state issues SNAP benefits to everyone on a single day, the remaining states have multiple issuance days, with 14 states issuing benefits to recipients over fewer than 10 days, 19 states have 10 issuance days, and less than 7 states distribute benefits over more than 10 days. In terms of public policy, our results suggest that having SNAP disbursement later in the month benefits low-income households in terms of reduced ER visits for childhood injuries.

## Data Availability

The data that support the findings of this study are available from the University of Missouri Center for Health Policy and the Missouri Department of Social Services but restrictions apply to the availability of these data, which were used under license for the current study, and so are not publicly available.

## Appendix

**Table 4:** Descriptive statistics

	All	Week of SNAP Disbursement		
		Week 1	Week 2	Week 3
	Mean	Mean	Mean	Mean
Injury-Related ER Claims	0.202	0.201	0.203**	0.203
Female	0.462	0.467	0.459***	0.459***
White	0.598	0.590	0.608***	0.597***
Black/African American	0.321	0.328	0.313***	0.323***
Hispanic	0.067	0.065	0.068***	0.069***
Age	2.454	2.441	2.450**	2.471***
HH Size	3.419	3.448	3.413***	3.398***
SNAP Amount	467.936	472.318	467.000***	464.618***
Observations	1288,552	419,676	438,145	430,731

Asterisks indicate whether the mean is statistically different from Week 1 (*** p <0.01, ** p <0.05, * p <0.1)

Source: Authors’ analysis of data from Missouri Department of Social Services. Sample of ER claims for children (0-5 years), from 2010-2013.

## Abbreviations

ER: Emergency room
SNAP: Supplemental Nutrition Assistance Program
USDA: United Stated Department of Agriculture

## Footnotes

1 We also included additional codes 7810–7814 and 7818, which indicate abusive head trauma conditions. These additional codes added < 3000 observations.

2 The importance of SNAP benefits to the household food supply over the month could be a function not only of when SNAP benefits are received but also of the size of the SNAP benefit. To explore the possible relationship between the size of SNAP benefits and the probability of ER claims related to childhood injuries, out of the universe of all Medicaid ER claims for children in Missouri jointly participating in the Medicaid and SNAP programs, in results not shown, we estimated probit models that include the size of the SNAP benefit, while controlling for the full set of covariates indicated in equation 1. We find a positive relationship between ER visits and the generosity of the benefit. But in contrast to other health outcomes [16, 17], there is no statistical difference between the size of the association at different levels of SNAP benefits.

3 Our main results are robust to different specifications of age as well as splitting the sample by age (0–3 and 4/5). When we add year dummy variables to the model with the birth year as the reference group, they are statistically significant and positive indicating that the risk of childhood injuries increases across the early childhood period after the birth year.
